# Adipose tissue–brain crosstalk in comorbid obesity and traumatic brain injury: Insights into mechanisms

**DOI:** 10.4103/NRR.NRR-D-25-00023

**Published:** 2025-03-25

**Authors:** Susan C. Burke, Bogdan A. Stoica, Rebecca J. Henry

**Affiliations:** UCD School of Biomolecular and Biomedical Science, UCD Conway Institute, University College Dublin, Dublin, Ireland; Department of Anesthesiology and Shock, Trauma and Anesthesiology Research (STAR) Center, University of Maryland School of Medicine, Baltimore, MD, USA

Obese individuals who subsequently sustain a traumatic brain injury (TBI) exhibit worsened outcomes including longer periods of rehabilitation (Eagle et al., 2023). In obese individuals, prolonged symptomology is associated with increased levels of circulatory pro-inflammatory markers up to 1 year post-TBI (Eagle et al., 2023). Despite this, the mechanisms driving worsened outcomes remain poorly understood. Expanding our understanding of the underlying mechanisms driving obesity-induced exacerbations of TBI deficits is important at a fundamental physiological level and for the identification of novel therapeutic approaches for TBI patients with underlying metabolic dysfunction.

Both TBI (Faden et al., 2021) and obesity (Cope et al., 2018; Guo et al., 2020) are characterized by peripheral and central inflammation, driven, at least in part, by alterations in activation states of macrophages and microglia, respectively. This neuro- and systemic- inflammation has been linked to neurobehavioral and cognitive dysfunction in TBI (Henry et al., 2020) and obesity (Cope et al., 2018). In pre-clinical models of comorbid diet-induced obesity and TBI, obesity leads to an amplification of TBI-related microglial activation (Sherman et al., 2016) and cognitive decline (Ibeh et al., 2023). Under obese states, adipose tissue macrophages account for approximately 50% of the cell population in the visceral adipose tissue (VAT) (Russo and Lumeng, 2018). Adipose tissue macrophages release pro-inflammatory mediators into the circulation that in turn contribute to low-grade chronic inflammation (Kane and Lynch, 2019) and subsequent neuroinflammation. A key family of inflammatory mediators implicated in obesity-induced inflammation is the NOD-like receptor protein 3 (*NLRP3*) inflammasome complex. Notably, obesity-induced VAT NLRP3 inflammasome activation is reported to mediate neuroinflammation and subsequent cognitive decline via activation of the microglial interleukin-1 receptor 1 (IL-1R1) (Guo et al., 2020).

Notably, TBI-induced peripheral inflammation is suggested to exacerbate secondary neuroinflammatory responses, subsequent neurodegeneration, and neurological decline (Faden et al., 2021). Thus, it is plausible to suggest that there is, in the context of TBI, a bidirectional neuroimmune relationship between the brain and periphery, which may be targeted therapeutically. Accordingly, our recent preclinical study hypothesized that there is a bi-directional neuroimmune relationship between the brain and adipose tissue in the presence of comorbid diet-induced obesity and TBI (Henry et al., 2024).

**Figure 1:**
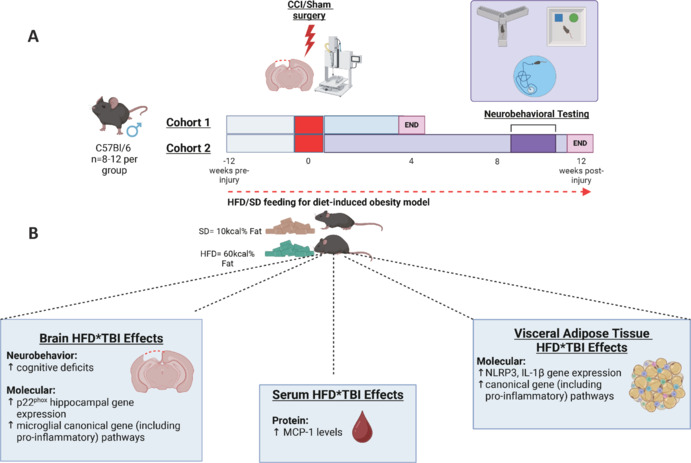
Interaction of sustained HFD feeding and experimental TBI alters VAT, serum, and brain immune microenvironment; effects of which are associated with an exacerbation of cognitive dysfunction. (A) Experimental design (Henry et al., 2024). Adult male C57Bl/6 mice were placed on either HFD (60% kcal) or SD (10% kcal) for 12 weeks prior to induction of either CCI or Sham surgery. The diet continued for the duration of the study. Cohort 1 was sacrificed at 28 dpi with VAT, brain tissue, and serum samples collected for RNA/protein analysis. Cohort 2 underwent a battery of neurobehavioural cognitive tests through 70–84 dpi, including the Y-Maze, NOR, and MWM tests. Tissue was collected at 90 dpi for transcriptomic analysis, with hippocampal and cortical CD11b^+^ cells isolated, RNA extracted, and analyzed using a Nanostring Glial Panel. RNA was also extracted from the VAT and analyzed using a Nanostring Neuroinflammation panel. Serum protein levels were measured using ELISA Kits. (B) Main findings (Henry et al., 2024). VAT-Molecular: Quantitative reverse transcription-polymerase chain reaction analysis showed increased gene expression of NOD-like receptor protein 3 (*NLRP3*) and interleukin *(IL)-1*β in HFD-TBI mice at 28 dpi, *versus* SD-TBI and HFD-Sham (*NLRP3*) counterparts. Ingenuity Pathway Analysis pathway analysis showed an HFD-TBI increase in the canonical gene (including pro-inflammatory) pathways compared to HFD-Sham counterparts at 90 dpi. Serum-Protein: Circulating levels of inflammatory mediator, MCP-1, was increased in HFD-TBI mice at 90 dpi, compared to SD-Sham. Brain- Neurobehavior: HFD-TBI mice exhibited worsened cognitive deficits in the NOR and MWM tests. HFD-TBI mice spent significantly less time with the novel object compared to SD-TBI mice in the NOR test. In the MWM, HFD-TBI mice increasingly utilized a random search strategy compared to other groups. Molecular: Quantitative reverse transcription-polymerase chain reaction analysis of hippocampal tissue showed increased gene expression of *p22*^*phox*^ in HFD-TBI mice compared to SD-TBI counterparts at 28 dpi. Ingenuity Pathway Analysis pathway analysis showed an HFD-TBI increase in microglial canonical gene (including pro-inflammatory) pathways compared to SD-TBI at 90 dpi. Created with BioRender.com. ♂; Male. CCI: Controlled cortical impact; dpi: days post-injury; HFD: high fat diet; IL: interleukin; MCP: monocyte chemoattractant protein; MWM: Morris Water Maze; NLRP3: NOD-like receptor protein 3; NOR: Novel Object Recognition; SD: standard diet; TBI: traumatic brain injury; VAT: visceral adipose tissue.

To test the hypothesis, adult male C57Bl/6J mice were fed either a standard diet (SD) (10 %kcal) or a high-fat diet (HFD) (60 %kcal) for 12 weeks before induction of experimental TBI, namely controlled cortical impact or sham surgery. Mice were followed for either 28 or 90 days post-injury (dpi) and the effects of TBI and sustained HFD feeding alone and in combination on VAT and systemic circulation inflammatory responses were examined. Additionally, chronic microglial inflammatory responses and cognitive function were investigated (**Figure 1A**).

In the VAT, although sustained HFD feeding alone led to an increase in CD45^+^ and CD11b^+^ cell numbers, this was further increased in the presence of TBI at 28 dpi (**Figure 1B**). Notably, increased immune cell numbers were associated with amplified phagocytic activity. While these findings suggest that adipose tissue macrophages/myeloid cells play a role in driving changes in the VAT inflammatory microenvironment, additional mechanistic studies are required to fully elucidate the relative role of the myeloid cell populations (including infiltrating neutrophils) in driving reported changes.

Further support for alterations in the VAT immune microenvironment was demonstrated through quantitative reverse transcription-polymerase chain reaction analysis, which revealed that while sustained HFD feeding was the primary driver of changes, TBI resulted in a significant exacerbation of HFD-induced increases in VAT expression of *IL-1*β and *NLRP3*, at 28 dpi. Transcriptomic analysis of more chronic changes (90 dpi) revealed that sustained HFD feeding resulted in a significant increase in gene expression of markers associated with the disease-associated macrophages subpopulation. Furthermore, IPA pathway analysis revealed that while sustained HFD feeding was the primary driver of changes in the VAT, both TBI and HFD-TBI resulted in notable alterations including amplification of canonical inflammatory pathways (**Figure 1B**).

Exploration of the inflammatory profile in the circulation revealed that although sustained HFD feeding alone failed to increase circulating levels of monocyte chemoattractant protein-1 at 90 dpi, HFD in the presence of TBI resulted in a significant increase (**Figure 1B**). Furthermore, leptin, an important adipokine in periphery-brain communication, was increased in the serum of both HFD and TBI, albeit to a lesser extent in the latter, at 28 dpi. Thus, these findings provide evidence for a potential role for selective inflammatory markers in driving peripheral-brain communication in the presence of co-morbid TBI and obesity.

Although sustained HFD feeding induced increases in hippocampal gene expression (*CD11b* and *p22*^*phox*^), TBI was shown to be the main driver, including evident TBI-induced increases in *IL-1*β, NOX-2, and *p22*^*phox*^, at 28 dpi. Notably, HFD exacerbated the TBI-induced increases in *p22*^*phox*^ selectively. To examine whether reported molecular changes were associated with alterations in neurobehavioural outcomes, selective cognitive testing was carried out between 70-84 dpi. Although TBI was shown to be the main driver of deficits in hippocampal-dependent working memory (Y-maze); sustained HFD feeding exacerbated TBI-induced deficits in non-spatial hippocampal-mediated memory (novel object recognition task; 77-78 dpi). Furthermore, HFD feeding exacerbated TBI-induced hippocampal dysfunction and spatial memory impairments (Morris water maze; 80–84 dpi). Collectively, these findings demonstrate that TBI results in chronic deficits in cognitive function, the effects of which were selectively exacerbated by sustained HFD feeding (**Figure 1B**).

To further delve into the cellular responses, we isolated CD11b^+^ cell populations from the perilesional cortex and hippocampus at 90 dpi. Unsurprisingly, TBI had the most evident impact on microglial transcriptional changes (**Figure 1B**). Specifically, TBI induced an increase in gene expression of markers associated with disease-associated macrophages, pro-inflammatory, and autophagy microglial phenotypes. Conversely, TBI resulted in a decrease in the expression of markers associated with the homeostatic microglia phenotype at 90 dpi. In a similar manner to analysis at the level of the VAT, IPA analysis revealed a significant interaction effect of sustained HFD feeding and TBI on multiple microglial reactive inflammatory pathways (**Figure 1B**). Overall, these findings demonstrate that TBI is associated with chronic alterations in microglial activation states (homeostatic → activated). This may drive, at least in part, the priming of an inflammatory environment that is vulnerable to insults including sustained HFD feeding.

In addition to the examination of microglia populations, we investigated the impact of TBI and HFD on gene expression changes in non-microglia cell populations including astrocytes and neurons. TBI downregulated genes associated with neuronal responses and neurogenesis, and upregulated genes associated with astrocyte and oligodendrocyte cell signatures. Notably, HFD feeding resulted in a decrease in gene expression of markers associated with a neuronal signature.

In summary, comorbid sustained HFD feeding and TBI amplify inflammatory responses, both in the periphery and brain, which in turn creates a self-propagating secondary injury loop exacerbating neurological and cognitive dysfunction (Henry et al., 2024). Pharmacological targeting of this bi-directional VAT-brain neuroimmune signaling axis may offer novel therapeutic approaches for TBI patients with pre-existing metabolic dysfunction.
